# Differential expression and co-expression reveal cell types relevant to genetic disorder phenotypes

**DOI:** 10.1093/bioinformatics/btae646

**Published:** 2024-10-28

**Authors:** Sergio Alías-Segura, Florencio Pazos, Monica Chagoyen

**Affiliations:** Computational Systems Biology Group, Centro Nacional de Biotecnología (CNB-CSIC), Madrid, 28049, Spain; Department of Molecular Biology and Biochemistry, Science Faculty, University of Málaga, Málaga, 29071, Spain; Computational Systems Biology Group, Centro Nacional de Biotecnología (CNB-CSIC), Madrid, 28049, Spain; Computational Systems Biology Group, Centro Nacional de Biotecnología (CNB-CSIC), Madrid, 28049, Spain

## Abstract

**Motivation:**

Knowledge of the specific cell types affected by genetic alterations in rare diseases is crucial for advancing diagnostics and treatments. Despite significant progress, the cell types involved in the majority of rare disease manifestations remain largely unknown. In this study, we integrated scRNA-seq data from non-diseased samples with known genetic disorder genes and phenotypic information to predict the specific cell types disrupted by pathogenic mutations for 482 disease phenotypes.

**Results:**

We found significant phenotype-cell type associations focusing on differential expression and co-expression mechanisms. Our analysis revealed that 13% of the associations documented in the literature were captured through differential expression, while 42% were elucidated through co-expression analysis, also uncovering potential new associations. These findings underscore the critical role of cellular context in disease manifestation and highlight the potential of single-cell data for the development of cell-aware diagnostics and targeted therapies for rare diseases.

**Availability and implementation:**

All code generated in this work is available at https://github.com/SergioAlias/sc-coex

## 1 Introduction

Rare diseases, despite their low individual prevalence, collectively affect millions of people worldwide. These conditions often stem from specific genetic mutations that disrupt normal cellular functions, resulting in diverse phenotypic manifestations across various organs and tissues. Each disease presents unique challenges in diagnosis and treatment, often resulting in prolonged patient suffering and substantial healthcare burdens.

Phenotypic manifestations, or phenotypes, are crucial for the clinical characterization and diagnosis of rare diseases. Due to the low prevalence of these conditions, phenotypes play a vital role in accurate diagnosis and patient stratification (Marwaha *et al.* 2022). From a systemic perspective, leveraging these phenotypes aids in prioritizing patient variants (Kelly *et al.* 2022) and developing phenotype-aware network-based approaches (Ranea *et al.* 2022). Disease phenotypes are reflected at the molecular network level to the same extent as diseases (Chagoyen and Pazos 2016). Consequently, approaching pathologies from a phenotypic point of view is especially useful in the case of rare diseases

Identifying the specific cell types impacted by genetic alterations is fundamental to understand the pathophysiology of diseases. Traditional tissue-level analysis may overlook critical cellular heterogeneity and the distinct roles that different cell types play in disease manifestation. Advances in single-cell technologies provide unprecedented opportunities to approach these problems at the cellular level. Mapping the cellular landscapes altered by disease-causing mutations allows for a more nuanced understanding of disease mechanisms.

The advent of single-cell RNA sequencing (scRNA-seq) (Tang *et al.* 2009) has revolutionized the study of diseases at the individual cell level. Increasingly, scRNA-seq data are utilized to study disease mechanisms (Hu *et al.* 2020, Khan *et al.* 2020, Auerbach *et al.* 2021, Dobie *et al.* 2022, Öz *et al.* 2022) and to predict disease phenotypes by comparing disease and control samples (Mao *et al.* 2024). These data also support systemic approaches to identify cell types relevant to disease states, as demonstrated by studies integrating scRNA-seq with GWAS data to infer cellular types involved in common diseases (Jagadeesh *et al.* 2022, Jia *et al.* 2022).

For several well-studied rare diseases, the specific cell types disrupted by genetic alterations are known. For instance, amyotrophic lateral sclerosis is a rare neurodegenerative disease that affects motor neurons in the brain and spinal cord leading to muscle weakness and atrophy (Brown and Al-Chalabi 2017); retinitis pigmentosa is a group of rare genetic disorders that result in the breakdown and loss of photoreceptor cells in the retina, causing vision loss (Wright *et al.* 2010); neonatal diabetes mellitus, a rare form of diabetes diagnosed in the first six months of life, involves dysfunctional insulin production by pancreatic beta cells (De Franco 2021); and severe combined immunodeficiency is a rare genetic disorder characterized by a severely compromised immune system due to disturbed development of functional T cells and B cells, leading to increased susceptibility to infections (van der Burg and Gennery 2011). However, the cell types underlying most rare disease manifestations are largely unknown. To bridge this gap, the pioneer work of Hekselman *et al.* (2024) predicted cell types associated with several Mendelian diseases based on their associated genes and their specific expression in scRNA-seq data from non-diseased tissues. They found 18% of known associations analyzed to be significant while providing novel putative associations.

In this work, we aim to predict the specific cell types disrupted by pathogenic mutations for a large number of genetic disorder phenotypes, instead of diseases, by integrating scRNA-Seq data with known genetic disorder genes and phenotypic information. As in Hekselman *et al.* (2024), our analysis uses single-cell data from non-diseased tissues due to the limited availability of scRNA-seq data from rare disease patients, attributed to cost, technical limitations, and sample acquisition challenges. Known causative mutations of genetic disorders typically originate from germline mutations but manifest clinically in specific organs or tissues. Various molecular mechanisms, such as tissue differential gene expression and tissue-specific protein interactions, can explain these tissue-specific manifestations of the same genotype (Barshir *et al.* 2014, Feiglin *et al.* 2017, Hekselman and Yeger-Lotem 2020, Sharon *et al.* 2022). Here, we investigate two mechanisms at the cell type level: differential expression (previously explored by (Hekselman *et al.* 2024) in the context of cell types and diseases) and co-expression (which has not previously been explored in this context). Phenotype-related genes might exhibit higher levels of expression in the involved cell type compared to other cell types in the tissue, or be highly co-expressed among them in that cell type compared to other gene pairs, indicating a functional connection in the latter case. Comparing our results with documented phenotype-cell associations from the literature (Novoa *et al.* 2024), we found that differential expression analysis revealed 13% of known associations, while co-expression analysis elucidated 42%. Many of the other predicted putative associations can point to relationships between phenotypes and cell types not yet discovered that could be investigated.

## 2 Methods

### 2.1 Data

We obtained single-cell transcript counts (scRNA-seq) from 26 non-diseased tissues from the Human Phenotype Atlas (HPA) version 21 https://v21.proteinatlas.org/ with their corresponding cluster and cell-type annotations (Karlsson *et al.* 2021). We obtained aggregated transcripts per million (TPM) for each cluster as well, representing the whole pool of single cells assigned to that cluster.

We retrieved disease phenotypes and their corresponding gene annotations from the Human Phenotype Ontology (HPO) release 2022–02-14 (https://hpo.jax.org) (Robinson *et al.* 2008). We gathered anatomical entities such as organs, tissues, and body parts from the Uber-anatomy ontology (Uberon) release 2022-02-21 (https://www.ebi.ac.uk/ols4/ontologies/uberon) (Mungall *et al.* 2012).

We finally excluded four tissues from the analysis: one, bone marrow, due to not having associated phenotypes (there were no HPO terms associated with its Uberon terms) (see Mapping tissues to phenotypes); and three (rectum, lymph node and placenta) due to not having known phenotype-cell type associations (according to CoMent) (see Literature analysis). A total of 22 tissues were finally analyzed.

### 2.2 Mapping tissues to phenotypes

In order to map tissues to phenotypes, we used the information provided by the HPO. We proceeded as follows: first, we manually assigned the Uberon term that corresponds to each tissue analyzed (Supplementary Table S1). We then retrieved all the descendant terms for each tissue term from the Uberon .obo file. This provided a list of anatomical terms (Uberon) for each tissue. Then, for each Uberon term we obtained associated HPO phenotype terms from the HPO .owl file. For each of these HPO terms, we obtained their descendant terms from the HPO .obo file. This provided the final list of phenotype terms (HPO) for each tissue.

Finally, we obtained genes for each phenotype (HPO term) to analyze from the annotation file phenotype_to_genes.txt. We kept only sufficiently informative and specific HPO terms for further analysis: those with at least 20 associated genes and no descendant terms with more than 20 associated genes. This threshold is arbitrary, but close to the one established (25 genes) in our previous work that measured the modularity of phenotypes in the human interactome (Chagoyen and Pazos 2016). We performed Ensembl ID- Entrez ID conversion using BioMart (Kinsella *et al.* 2011).

### 2.3 Computing differential expression and co-expression values

We calculated two metrics for each phenotype in each cell cluster (per tissue) based on: differential gene expression and co-expression.

#### 2.3.1 Differential expression

The HPA dataset aggregated by cluster was used to calculate the differential expression. This approach minimizes the problem of technical noise (zero-counts) in scRNA-seq data (Karlsson *et al.* 2021). The dataset contains, for each gene and cluster, a TPM value. We computed the fold change (logFC), according to the following formula:

(1) $$\log\mathrm{FC}=\log\left(\frac{{\mathrm{TPM}}_{\mathrm{cluster}}}{{\underline{\mathrm{TPM}}}_{\mathrm{background}}}\right)$$

Where *TPMcluster* is the TPM value for the gene of interest in the cluster of interest, and ${\underline{\mathrm{TPM}}}_{\mathrm{background}}$ is the average TPM value for the gene of interest in the rest of the clusters of the dataset.

#### 2.3.2 Co-expression of gene pairs

The HPA scRNA-seq dataset was used to calculate gene co-expressions. For each cell cluster in a tissue, we calculated all gene pair co-expressions with COTAN (COexpression Tables ANalysis) (Galfrè *et al.* 2021), a method specifically designed to work with the characteristics and limitations of scRNA-seq data. In a pre-processing step, we first removed cell outliers and performed data quality checking adapting the vignette provided by COTAN authors (see Availability for details). To generate co-expression matrices, for each pair of genes we built a 2x2 contingency table containing the number of cells in each possible condition (expressing both genes, only the first, only the second or neither). With this table, we computed the GPA co-expression index (COEX) that measures the deviation of co-expression from the expected proportion under the independence assumption (ranging from −1 to 1). For a comprehensive description of the co-expression index (COEX), see Galfrè *et al.* (2021). We calculated a total of 444 gene co-expression networks, one for each cell cluster.

### 2.4 Statistical assessment

For each cell cluster and phenotype pair within a tissue, we employed the Kolmogorov–Smirnov (K–S) test to determine whether genes associated with a phenotype have significantly higher differential expression (logFC) compared to the background distribution (logFC of the rest of genes) or extreme co-expression values (COEX) among them compared to the remaining co-expressions. We finally corrected these significance values (*P*-values) for multiple testing using the Benjamini–Hochberg false discovery rate (FDR) method.

Note that a tissue can have several cell clusters of the same cell type (e.g. tissue liver has five clusters annotated as hepatocytes). The final score for a cell type is the minimum FDR of all cell clusters annotated with that cell type (corresponding with the less restrictive approach, where a cell subtype is enough to justify a known phenotype-cell type association). We considered cell types associated with a phenotype if their minimum FDR < .001.

### 2.5 Literature analysis

We compiled a set of known phenotype-cell type relations from the literature. For that, we built a corpus of literature co-mentions between the phenotypes and cell-types analyzed in this work using CoMent (Novoa *et al.* 2024). CoMent computes the statistical significance of co-mentions of two concepts using the entire PubMed corpus. The method is described in detail in (Pazos *et al.* 2022). We considered known relations (positives) those phenotype-cell type pairs with a CoMent *P*-value < .001, and unknown (negatives) those that are not found co-mentioned in the literature or are co-mentioned with a *P*-value >= .001.

## 3 Results

**Figure 1.**
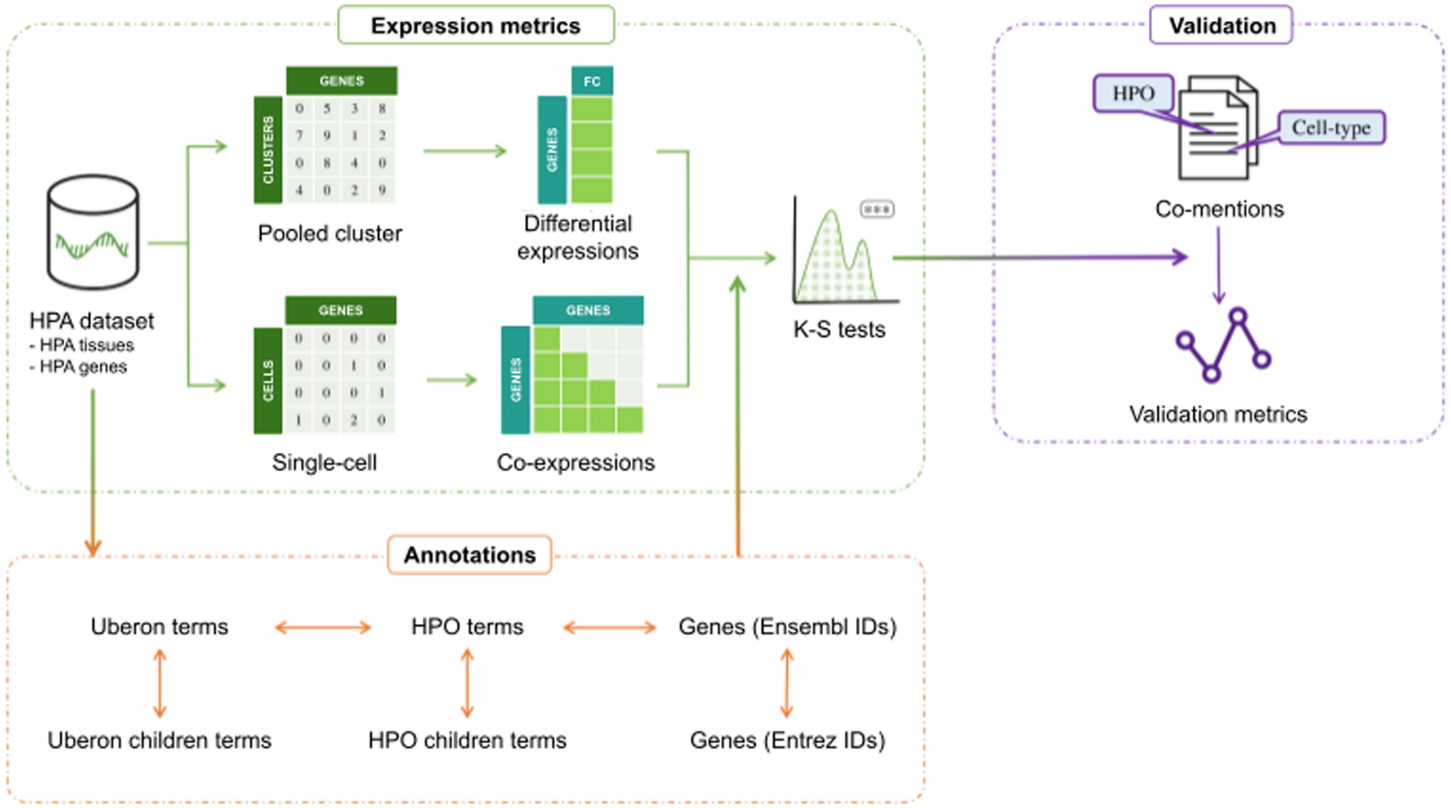
Overview of the analysis. We selected 482 non-redundant genetic disease phenotypes for 22 tissues based on their anatomical locations in the HPO and Uberon ontologies. We used non-diseased single-cell RNA-seq data from the Human Protein Atlas (HPA) to compute the differential expression and co-expression of phenotype genes for each cell cluster in the corresponding tissue. We performed Kolmogorov–Smirnov statistical tests and applied FDR correction to assign cell types to phenotypes. We compared our results with a set of phenotype-cell type associations compiled from the literature.

We obtained single-cell RNA-seq data from non-diseased human tissues and their cell cluster annotations from the Human Protein Atlas (HPA) (Karlsson *et al.* 2021). We then computed gene differential expression and co-expression of all gene pairs for each cell cluster within a tissue. For each tissue, we selected relevant phenotypes for analysis based on their anatomical locations according to the HPO (Robinson *et al.* 2008). We obtained the set of genes associated with a given phenotype from the HPO. For each cell cluster within a tissue, we performed Kolmogorov–Smirnov statistical tests and corrected for multiple testing to determine the extent to which the differential expression and co-expression of genes associated with a phenotype significantly differ from the background distribution of the remaining differential expression and co-expression of genes not associated with the phenotype in the cell cluster of interest. Finally, we compared our results with a set of phenotype-cell type associations compiled from the literature. See Methods for details and Fig. 1 for an overview of the analysis.

### 3.1 Cell-type analysis

We analyzed scRNA-seq data from 22 tissues from different studies (Hawrylycz *et al.* 2012, Chen *et al.* 2018, Guo *et al.* 2018, Henry *et al.* 2018, MacParland *et al.* 2018, Menon *et al.* 2019, Parikh *et al.* 2019, Vieira Braga *et al.* 2019, De Micheli *et al.* 2020, He *et al.* 2020, Liao *et al.* 2020, Lukassen *et al.* 2020, Man *et al.* 2020, Qadir *et al.* 2020, Solé-Boldo *et al.* 2020, Wang *et al.* 2020a, Wang *et al.* 2020b, Wang *et al.* 2020c, Bhat-Nakshatri *et al.* 2021, Hildreth *et al.* 2021). These data were compiled by the Human Protein Atlas (HPA) and was annotated with 444 cell clusters corresponding to 78 distinct cell-types (Karlsson *et al.* 2021). After mapping anatomical terms and corresponding HPO terms (see Methods), we finally analyzed 482 non-redundant phenotypes (corresponding to the most specific terms in the HPO hierarchy within those with at least 20 genes). The distribution of the number of genes per phenotype is shown in Supplementary Fig. S1. The number of phenotypes per tissue finally analyzed is variable (Supplementary Fig. S2). A total of 3338 phenotype-cell type pairs were analyzed (see Supplementary Table S2 for results).

**Figure 2.**
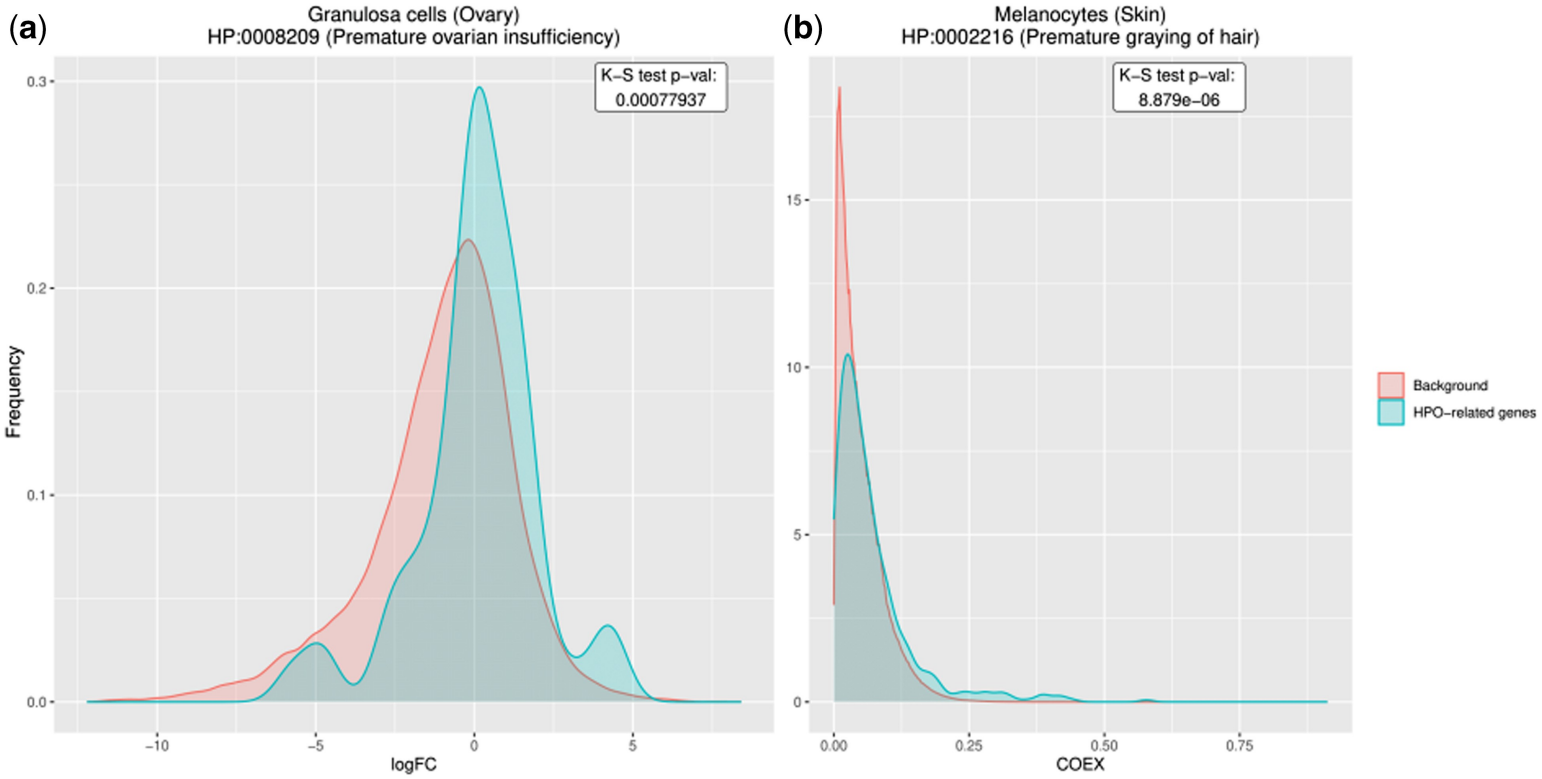
Examples of significant phenotype—cell type associations based on differential expression and co-expression. (a) Distributions of FC values of genes associated with and not associated with the 'premature ovarian insufficiency' phenotype in granulosa cells from the ovary. The phenotype-cell type association is significant (genes are differentially expressed, *P*-value = .00078). (b) Distributions of co-expression values of genes associated with and not associated with the 'premature graying of hair' phenotype in melanocytes from the skin. The phenotype–cell type association is significant (there is higher gene co-expression, *P*-value = 8.879e-6).

Based on differential expression, we found significant associations (FDR < 0.001) for 202 phenotype-cell type pairs corresponding to putative associations. For example, granulosa cells (GCs) from ovary tissue were significantly associated with premature ovarian insufficiency (POI) (Fig. 2a). This result is relevant as GCs surrounding oocytes play a pivotal role in folliculogenesis, emerging as an important etiological factor in POI (Liu *et al.* 2023).

Based on co-expression, we found significant associations (FDR < 0.001) for 1047 phenotype-cell type pairs. As an example, melanocytes from skin tissue were significantly associated with premature graying of hair (Fig. 2b). Melanocytes are responsible for producing melanin, the pigment that gives hair its color. Both oxidative stress and genetic factors contribute to the premature graying of hair by impairing melanocyte function (Shi *et al.* 2014).

To check whether they offer similar or complementary explanations, we next compared the results obtained by differential expression and co-expression approaches (Supplementary Fig. S3). A large number of the phenotypes analyzed (404 of 491, 82.3%) have no significant association by differential expression analysis. In contrast, we found at least one significant association for a higher number of phenotypes (317 of 491, 64.6%) by co-expression analysis. Only 177 phenotype-cell type pairs were predicted by both differential expression and co-expression (corresponding to 79 distinct phenotypes, with sizes ranging from 21 to 168 genes). For example, proximal tubular cells are significantly associated with proximal tubulopathy (the dysfunction of the proximal tubule, the portion of the duct system of the nephron of the kidney which leads from Bowman's capsule to the loop of Henle, according to HPO definition), by both differential expression and co-expression. Notably, 88% of the phenotype-cell type putative associations predicted by differential expression were also predicted by co-expression.

To assess whether there is a bias in predicting phenotype-cell type associations based on phenotype size (i.e. the number of associated genes), we compared the distributions of significant versus non-significant associations across both metrics. Significant differences in phenotype size were observed for both differential expression (*P*-value = 3.16e-19) and co-expression (*P*-value = 2.86e-84), with a tendency to predict larger phenotypes in both cases. Additionally, there is a marginally significant difference in size for the putative associations found by differential expression and co-expression (*P*-value = .0481). Significant differential expression associations tend to be of larger phenotypes than significant co-expression associations.

### 3.2 Literature analysis

To evaluate the relevance of differential expression and co-expression as mechanisms to account for cell type specific manifestations of diseases, we compiled a set of known phenotype-cell type associations from the literature following a previously described methodology (CoMent) (Novoa *et al.* 2024). A total of 541 of phenotype—cell type pairs from our set were found significantly co-mentioned in the literature (CoMent *P*-value < .001) and were used as a set of known (positive) associations, while the remaining pairs were treated as negatives. No bias was observed when comparing the size distribution of the phenotypes with at least one cell type association in the literature (used for validation) to those without (*P*-value = .1029).

We selected 482 non-redundant genetic disease phenotypes for 22 tissues based on their anatomical locations in the HPO and Uberon ontologies. We used non-diseased single-cell RNA-seq data from the Human Protein Atlas (HPA) to compute the differential expression and co-expression of phenotype genes for each cell cluster in the corresponding tissue. We performed Kolmogorov–Smirnov statistical tests and applied FDR correction to assign cell types to phenotypes. We compared our results with a set of phenotype-cell type associations compiled from the.

Globally, differential expression captured 12.75% of the known phenotype-cell type associations, correctly predicting 95.14% of the negative associations. Co-expression, captured a larger set of associations (41.59%), at the expense of a lower proportion of correctly predicted negative associations (72.37%). For a complete summary table with prediction metrics, see Supplementary Table S3.

Some of the putative associations revealed in our analysis might be still relevant even if we found no significant co-mentions in the literature. For example, Langerhans cells from skin are putatively associated with recurrent bacterial skin infections by both differential expression and co-expression. This association, although not significantly co-mentioned, has been described in previous studies (Rajesh *et al.* 2019). Basal keratinocytes are putatively associated with skin erosion by differential expression. These cells are known to possess properties of stem cells and are essential in maintaining the integrity of skin and damage recovery (Yin *et al.* 2023). And respiratory ciliated cells from bronchus are putatively associated with recurrent bronchitis according to co-expression analysis. It is known that recurrent bronchitis is associated with loss of ciliated cells in children (Gaillard *et al.* 1994).

Other predicted associations, not supported by previous publications, warrant further investigation. For example, according to our analysis, fibroblasts are putatively associated with various types of hernias (congenital diaphragmatic hernia, hiatus hernia, and inguinal hernia), squamous epithelial cells with tracheoesophageal fistula, inhibitory neurons with diffuse cerebral atrophy, and endothelial cells with retinal hemorrhage. The complete list of phenotype-cell type associations analyzed and corresponding results is provided in Supplementary Table S1.

## 4 Discussion

In this study, we performed an exploratory analysis to understand how differential expression and gene co-expression in non-diseased single cells could elucidate the mechanisms through which known genetic alterations disrupt specific cell types within a tissue, leading to abnormal phenotypes in genetic disorders. Our analysis encompassed 482 non-redundant phenotypes associated with 22 tissues, within the context of 444 single-cell clusters representing 78 distinct cell types. Differential expression and co-expression are distinct mechanisms and as such provide different results. Differential expression points to higher gene expression levels in those cell types involved in the abnormal phenotype and captures a limited proportion of known phenotype-cell type associations (13%). Meanwhile, co-expression accounts for a higher similarity in expression patterns across individual cells of the cell type involved in the phenotype, implying shared regulatory mechanisms or participation in common biological functions or processes. Co-expression revealed a higher proportion of known phenotype-cell type associations (42%). Additionally, most significant phenotype-cell type relations found by differential expression were also found to be significant by co-expression.

We focused on analyzing single-cell data from non-diseased samples, as these datasets provide a critical baseline to understand cellular heterogeneity and normal physiological states. Phenotypic information is especially relevant for the diagnosis of rare diseases patients. However, single-cell data from rare disease patients is still scarce. This is due to several challenges, including the difficulty in obtaining sufficient patient samples, high costs, and the technical and ethical complexities associated with single-cell sequencing in clinical settings. Consequently, non-diseased samples serve as a necessary proxy for their practical use in clinical applications.

Our primary objective was to identify the specific cell types within a tissue where a phenotype primarily manifests, not including cell types from other tissues in the analysis. We also excluded phenotypes arising from complex interactions among different tissues. For instance, abnormalities in body height, such as short or tall stature, can result from disruptions in cellular functions within both the skeletal and endocrine systems. To accomplish this, phenotypes not directly associated with at least one tissue (as defined by our Methods, see Mapping tissues to phenotypes) were excluded. Indeed, following our mapping strategy only nine phenotypes were analyzed in more than one tissue (Supplementary Table S4), such as hepatosplenomegaly, which is related to both the liver and spleen.

In some tissues, several cell clusters are annotated with the same cell type, indicating that their transcriptional profiles are sufficiently different. For example, in the liver there are five cell clusters annotated as hepatocytes. Our approach recognizes these differences as it analyzes clusters independently. For instance, 13 of the 26 brain cell clusters annotated as excitatory neurons are significantly associated with cortical dysplasia based on co-expression analysis, while the other 13 clusters are not. Similarly, in the lung, one of the two clusters annotated as macrophages is significantly associated with pleural effusion, whereas the other is not. This illustrates the potential of our approach to go below the cell type level to allow, e.g. the exploration of specific subtypes affected by the genetic variants.

We acknowledge that not all genes may be equally relevant for a phenotype, as some phenotypes may only occasionally occur in a disease or may be secondary to the primary disease cause. However, in this study, we treated all genes equally due to the limited availability of phenotype frequency data for most diseases.

Notably, 56.93% (308 of 541) of phenotype-cell type relations extracted by literature co-mentions could not be captured by either differential expression or co-expression data from healthy tissues. There are several possible explanations for that. Phenotype manifestations often result from complex molecular mechanisms that are not captured by transcriptomic data alone like post-translational modifications, protein–protein interactions, or epigenetic changes. A phenotype might be associated with transient gene expression changes during specific developmental stages or in response to environmental stimuli. Single-cell RNA-seq data from adult tissues at a single time point may not capture these temporal dynamics. Technical limitations of scRNA-seq, such as dropout events where certain transcripts are not detected, and biases in sequencing depth, can affect the reliability of differential expression and co-expression analyses. Some phenotypes are influenced by interactions among multiple cell types within the same tissue. These multifactorial interactions can obscure clear associations between specific genes and cell types. Notably, 27% of the analyzed phenotypes have more than one cell type documented in the literature. Our approach captured the involvement of multiple cell types in certain phenotypes (Supplementary Fig. S4). For example, dilated cardiomyopathy is significantly associated with both cardiomyocytes and fibroblasts in the literature (Tsuru *et al.* 2023) and both cell types were significant in our differential expression and co-expression analyses. However, in some cases, partial involvement of a subset of genes in each cell type might not yield significant results for each cell type independently.

The phenotype-cell type relationships we compiled from the literature may not encompass the complete set of known associations. Our large-scale co-mention analysis has certain limitations. Polysemic words can result in incorrect inferences. Additionally, relevant relationships may be missed due to their presence in a limited number of articles, and certain terminologies might not have been considered in our searches. Despite these weaknesses, our dataset remains the most comprehensive available. To the best of our knowledge, there are no curated resources that systematically compile relationships between disease phenotypes and cell types.

Some of the relevant cell types might not be represented in the scRNA-seq data analyzed. According to our literature analysis 57% of phenotypes have no significant association with the cell types analyzed in this work. The percentage is exceptionally high in certain tissues, such as the brain where nearly 80% of them (69 of 87) have no significant association. For instance, arrhinencephaly is significantly associated with mitral cells by CoMent, a specific type of neuron of the olfactory system which is not represented in the brain cell clusters analyzed.

Despite these limitations, our results suggest that differential expression and co-expression inferred from non-diseased single-cell data can be utilized to explore potential cell types involved in the phenotypic manifestations of rare diseases. In a previous study, Hekselman *et al.* (2024) captured ∼18% of the known disease–cell type associations analyzed by an approach based on specific gene expression. In this work, our differential expression approach captured a slightly lower percentage (13%) of known phenotype-cell type associations, while pointing to higher co-expression as an additional mechanism relevant for predicting putative associations. Understanding the specific cell types affected by genetic variants is crucial for elucidating the molecular and cellular mechanisms underlying rare disease manifestations. This knowledge is essential for developing improved diagnostic tools and novel treatments or therapies targeting the relevant cell types. Diagnostics could focus on markers in these specific cell types instead of whole tissues or unspecific samples. Similarly, treatments could be specifically targeted to these cell types. This will ultimately advance personalized healthcare solutions for patients with rare diseases.

## Supplementary Material

btae646_Supplementary_Data
