# Supplementary material for: Differential expression and co-expression reveal cell types relevant to genetic disorder phenotypes
Source: Bioinformatics. 2024 Oct 28;40(11):btae646. doi: 10.1093/bioinformatics/btae646 (PMC11549017; doi:10.1093/bioinformatics/btae646)
Supplement: btae646_Supplementary_Data [file btae646_supplementary_data.zip › supplementary_material_final.pdf]

Supplementary material

This file contains supplementary tables and figures to the work 'Differential expression and co-expression reveal cell types relevant to genetic disorder phenotypes' by Alias-Segura *et al*. (2024), published in *Bioinformatics*.

Supplementary tables (Tables S1-S4)

Table S1. Manually assigned Uberon term for each tissue.
Table S2. Results for all phenotype-cell type pairs analyzed (see supplementary file)
Table S3. Prediction metrics.
Table S4. HPO terms analyzed in more than one tissue.

Supplementary figures (Figures S1-S4)

Figure S1. Distribution of phenotype sizes (number of genes per HPO term).
Figure S2. Number of HPO terms analyzed per tissue.
Figure S3. Comparison of differential expression (y-axis) and co-expression (x-axis) results: log-transformed adjusted p-values (FDR) for each phenotype-cell type pair analyzed.
Figure S4. Percentage of significant cell types per phenotype - tissue pair, from literature (CoMent), fold change (FC) and co-expression (COEX).

Supplementary tables (Tables S1-S4)

Table S1. Manually assigned Uberon term for each tissue.

Tissue (as in HPA dataset)	Uberon code	Tissue (as in Uberon)
Colon	UBERON:0001155	colon
Eye	UBERON:0000966	retina
Heart muscle	UBERON:0000948	heart
Small intestine	UBERON:0002108	small intestine
Kidney	UBERON:0002113	kidney
Liver	UBERON:0002107	liver
Lung	UBERON:0002048	lung
Placenta	UBERON:0001987	placenta
Prostate	UBERON:0002367	prostate gland
Rectum	UBERON:0001052	rectum
PBMC	UBERON:0000178	blood
Testis	UBERON:0000473	testis
Pancreas	UBERON:0001264	pancreas
Skin	UBERON:0002097	skin of body
Brain	UBERON:0000955	brain
Bronchus	UBERON:0002185	bronchus
Endometrium	UBERON:0001295	endometrium
Skeletal muscle	UBERON:0014892	skeletal muscle organ
Ovary	UBERON:0000992	ovary
Adipose tissue	UBERON:0001013	adipose tissue
Esophagus	UBERON:0001043	esophagus
Lymph node	UBERON:0000029	lymph node
Bone marrow	UBERON:0002371	bone marrow
Spleen	UBERON:0002106	spleen
Stomach	UBERON:0000945	stomach
Breast	UBERON:0000310	breast

Table S2. Results for all phenotype-cell type pairs analyzed (see supplementary file)

Table S3. Prediction metrics.

**Method**	**TP**	**TN**	**FP**	**FN**	**TPR**	**TNR**	**PPV**
Co-expression	225	1975	822	316	0.4159	0.7061	0.2149
Differential expression	69	2664	133	472	0.1275	0.9524	0.3416

TP: True Positives; TN: True Negatives; FP: False Positives; FN: False Negatives; TPR: True Positive Rate; TNR: True Negative Rate; PPV: Positive Predictive Value

Table S4. HPO terms analyzed in more than one tissue.

**HPO_code**	**HPO_name**	**tissues**
HP:0000824	Decreased response to growth hormone stimulation test	Blood, Brain
HP:0001433	Hepatosplenomegaly	Liver, Spleen
HP:0002036	Hiatus hernia	Skeletal muscle organ, Stomach
HP:0002837	Recurrent bronchitis	Bronchus, Lung
HP:0003758	Reduced subcutaneous adipose tissue	Adipose tissue, Skin
HP:0006579	Prolonged neonatal jaundice	Liver, Skin
HP:0007763	Retinal telangiectasia	Eye, Skin
HP:0008232	Elevated circulating follicle stimulating hormone level	Blood, Brain
HP:0011969	Elevated circulating luteinizing hormone level	Blood, Brain

Supplementary figures (Figures S1-S4)

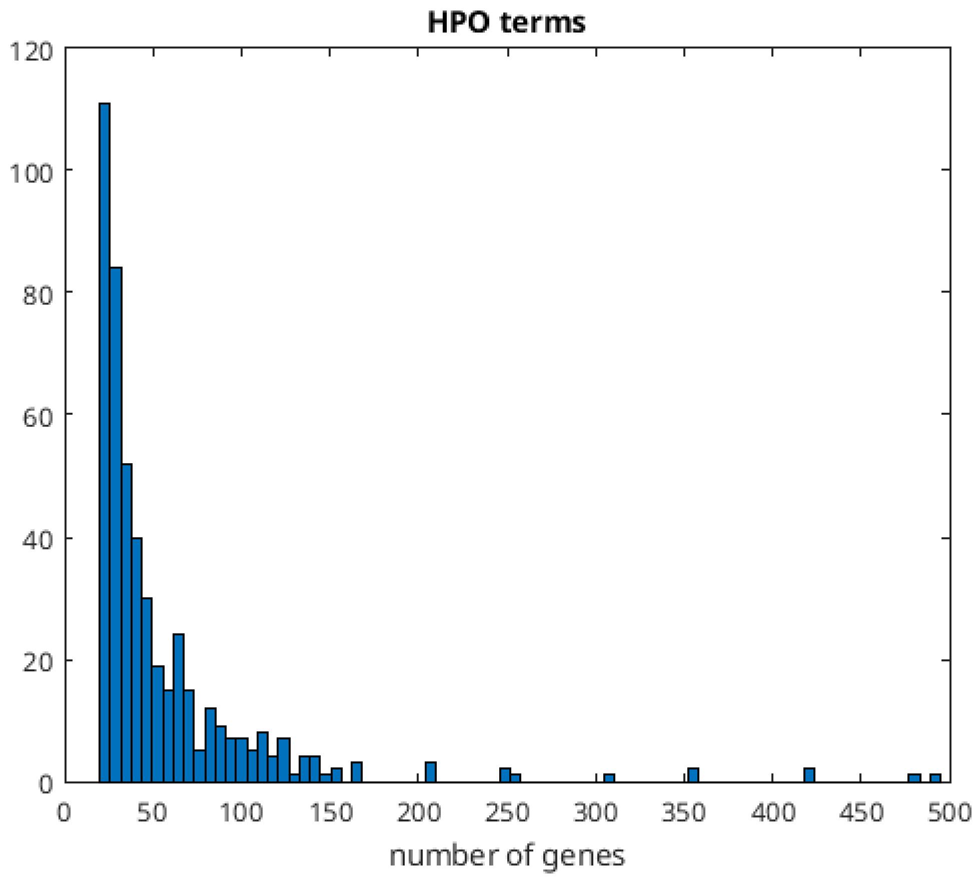

Figure S1. Distribution of phenotype sizes (number of genes per HPO term)

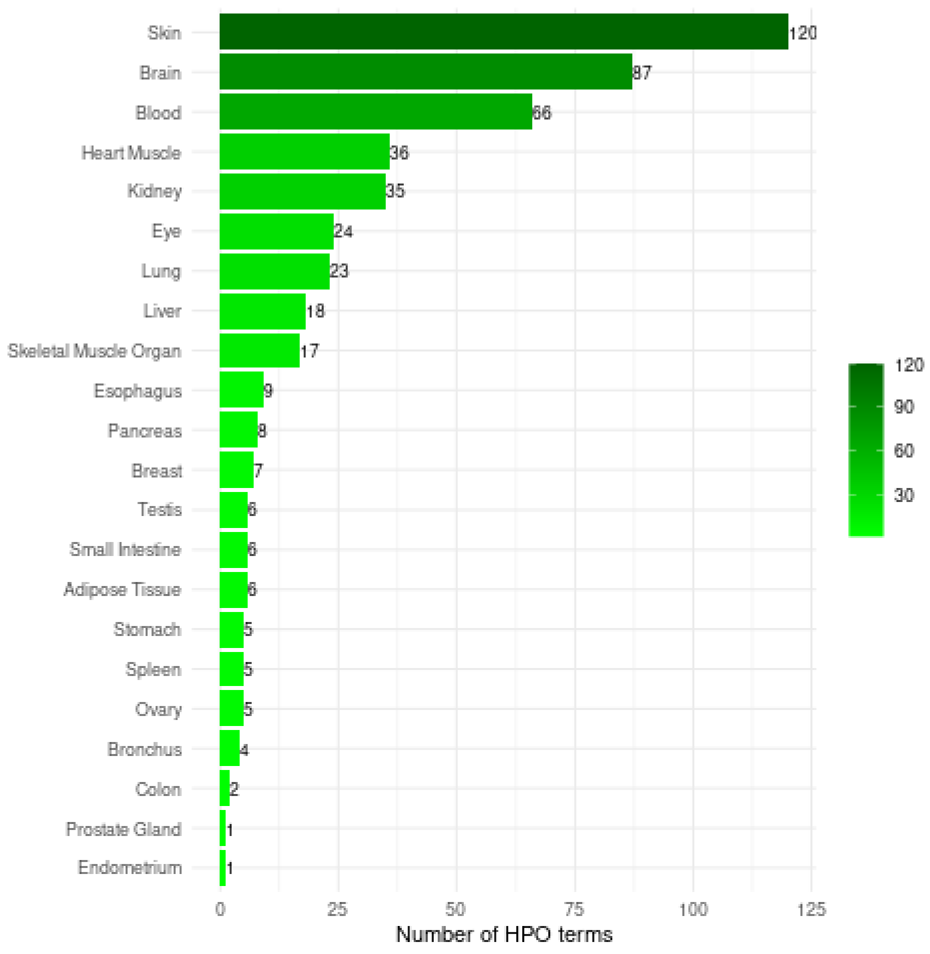

Figure S2. Number of HPO terms analyzed per tissue.

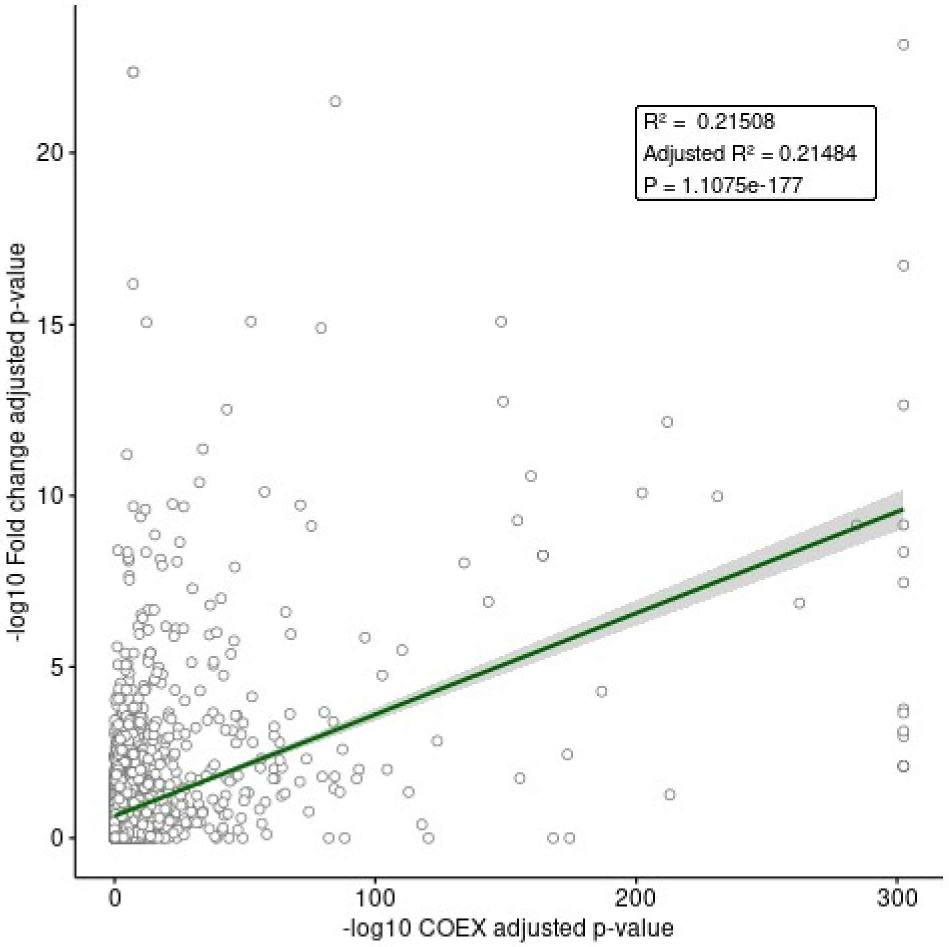

Figure S3. Comparison of differential expression (y-axis) and co-expression (x-axis) results: log-transformed adjusted p-values (FDR) for each phenotype-cell type pair analyzed.

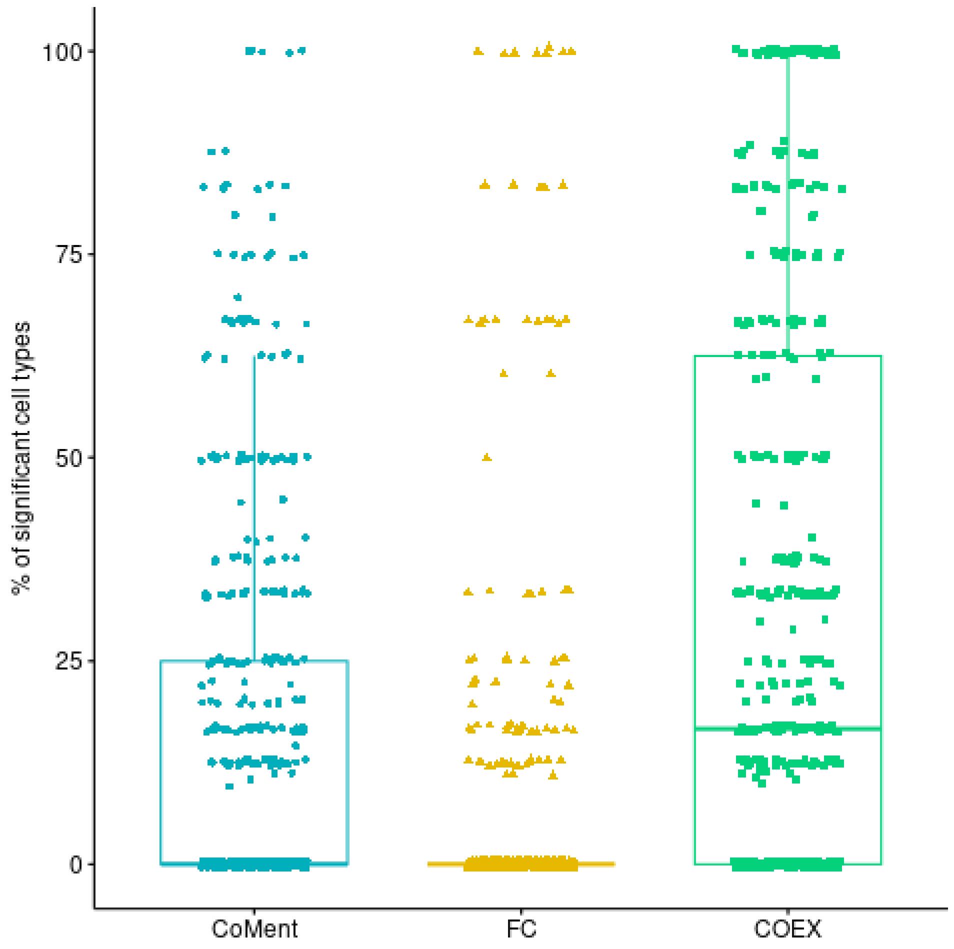

Figure S4. Percentage of significant cell types per phenotype - tissue pair, from literature (CoMent), fold change (FC) and co-expression (COEX).
